# Symptom clusters and sentinel symptoms in colorectal cancer patients during post-operative chemotherapy

**DOI:** 10.3389/fonc.2026.1808713

**Published:** 2026-06-30

**Authors:** Shengli Liu, Songyan Zhao

**Affiliations:** 1 School of Nursing, Affiliated Chifeng Clinical College of Inner Mongolia Medical University, Chifeng, Inner Mongolia, China; 2 Department of Nursing, Affiliated Chifeng Clinical College of Inner Mongolia Medical University, Chifeng Municipal Hospital, Chifeng, Inner Mongolia, China

**Keywords:** Adjuvant chemotherapy, colorectal cancer, longitudinal study, sentinel symptoms, symptom clusters

## Abstract

**Objective:**

This study aimed to identify the distribution characteristics, dynamic changes, and sentinel symptoms of symptom clusters in colorectal cancer patients undergoing post-operative adjuvant chemotherapy, providing a scientific basis for optimizing symptom management strategies.

**Methods:**

A prospective longitudinal study design was employed, using convenience sampling to recruit 220 colorectal cancer patients receiving their first post-operative chemotherapy from a tertiary hospital in Chifeng City, Inner Mongolia, from July 2024 to July 2025. The M.D. Anderson Symptom Inventory-Gastrointestinal Cancer Module (MDASI-GI) was used to assess symptoms after the 1st, 2nd, and 3rd chemotherapy cycles. Exploratory factor analysis was used to identify symptom cluster structures, generalized estimating equations were applied to analyze longitudinal changes in symptom severity, and the Apriori algorithm with association rule mining was employed to identify sentinel symptoms within each symptom cluster based on temporal precedence criteria. A total of 182 patients completed all follow-ups, with a loss-to-follow-up rate of 12.50% (calculated from 208 patients with valid T1 data).

**Results:**

Symptom burden increased progressively across chemotherapy cycles, with the average number of symptoms rising from 8.32 at T1 to 13.3 at T3. Fatigue, decreased appetite, and disturbed sleep were consistently the most prevalent symptoms, all reaching 96.7% by T3. Symptom cluster structure evolved from three clusters at T1 (gastrointestinal-nutritional, emotional-psychological, and sensory-neurological) to four clusters at T2 and T3, with the emergence of a distinct fatigue-pain cluster. All symptom severities increased significantly over time (P<0.001), with fatigue and constipation showing the most pronounced progression. Seven sentinel symptoms were identified across the three time points: in the gastrointestinal-nutritional cluster — taste changes (T1), nausea and diarrhea (T2), and abdominal bloating (T3); in the emotional-psychological cluster — disturbed sleep (T2), distress and sadness (T3).

**Conclusion:**

Colorectal cancer patients undergoing post-operative chemotherapy experience cumulative increases in symptom burden, with symptom cluster structures evolving dynamically throughout the treatment course. The identified sentinel symptoms represent potential monitoring targets that may inform proactive symptom management strategies, though further intervention studies are needed to validate their clinical utility.

## 1 Introduction

Post-operative adjuvant chemotherapy plays a crucial role in comprehensive cancer treatment by eliminating residual disease that could not be completely removed during surgery, reducing the probability of cancer cell metastasis to distant organs, improving patient survival rates, and significantly prolonging overall survival time (1–3). Despite its therapeutic benefits, adjuvant chemotherapy is frequently accompanied by a wide spectrum of treatment-related side effects that can substantially impact patients’ daily functioning and quality of life. Due to the diverse and often debilitating complications experienced by colorectal cancer patients during chemotherapy, the M.D. Anderson Symptom Inventory-Gastrointestinal Cancer Module (MDASI-GI) was specifically developed and validated to systematically quantify symptom severity and interference with daily activities. The MDASI-GI is a comprehensive, patient-reported outcome measure consisting of two distinct parts with 24 items total, covering both symptom severity and functional interference domains (4, 5). This validated instrument has demonstrated strong psychometric properties and cultural adaptability across diverse patient populations.

For colorectal cancer patients undergoing adjuvant chemotherapy, extensive research has documented that the most common and clinically significant symptom clusters are emotional-psychological symptoms (including anxiety, depression, and distress) and gastrointestinal symptoms (such as nausea, vomiting, diarrhea, and decreased appetite) (6, 7). These symptoms often co-occur and interact with each other in complex patterns. Epidemiological data reveal that symptom prevalence rates range widely from 5.4% to 85.8% depending on the specific symptom, assessment timing, and chemotherapy regimen used, with severity scores ranging from 0.51 ± 1.44 to 4.83 ± 3.23 on standardized scales. Notably, fatigue has been consistently identified as a cardinal symptom that is significantly associated with higher severity scores across multiple symptom dimensions (8).

Symptom clusters are conceptually defined as stable groups formed by multiple interrelated symptoms occurring simultaneously, sharing common underlying mechanisms or etiologies. These clusters represent more than mere symptom co-occurrence; they reflect interconnected pathophysiological processes. Accumulating research evidence consistently shows that symptom clusters have substantially more adverse impacts on treatment outcomes, clinical prognosis, functional status, overall quality of life, and healthcare resource utilization compared to individual symptoms experienced in isolation (9–13). The synergistic negative effects of clustered symptoms can create a downward spiral that compromises treatment adherence and recovery trajectories. In colorectal cancer survivors assessed during long-term follow-up, three distinct symptom clusters were clearly identified through statistical modeling approaches, with approximately 30% of survivors experiencing varying degrees of persistent physical or emotional-psychological symptoms that affect their daily activities and social reintegration, with chemotherapy exposure being identified as the primary influencing factor contributing to long-term symptom burden (14).

A comprehensive meta-analysis pooling data from 4,001 patients across multiple studies reported several common and clinically relevant symptom clusters including: (1) the affective-fatigue cluster comprising fatigue, anxiety, and depression; (2) the digestive system cluster including nausea, vomiting, and decreased appetite; (3) the pain-sleep disturbance cluster; and (4) the abdominal discomfort cluster characterized by abdominal pain and bloating (15). These findings highlight the multidimensional nature of symptom experiences and the need for holistic assessment strategies.

Accurate identification and characterization of symptom clusters helps healthcare professionals develop more targeted, efficient, and personalized symptom management plans that address the root causes rather than treating symptoms in isolation. However, despite growing recognition of symptom clusters’ clinical importance, consensus on selecting the most effective and strategic symptoms for clinical intervention remains notably lacking in current literature and clinical practice guidelines. To address this critical gap, the concept of ‘sentinel symptoms’ has recently emerged as a promising approach to managing complex symptom clusters more efficiently. Sentinel symptoms are operationally defined as key indicators within a symptom cluster — symptoms that appear earlier or demonstrate statistically stronger associations with other symptoms in the same cluster, thereby serving as potential early warning signals. By monitoring these sentinel symptoms, clinicians may be able to prioritize assessment efforts and potentially intervene before the full cascade of related symptoms develops. This approach is grounded in temporal association patterns rather than causal inference, and its clinical value requires further prospective validation. Recent longitudinal symptom network studies have demonstrated the value of examining dynamic, time-dependent relationships among symptoms. For example, Sun et al. (2026) applied cross-lagged panel network analysis to examine symptom dynamics in esophageal cancer patients during chemotherapy, identifying temporally influential symptoms that shaped the trajectory of other co-occurring symptoms (16). Such work provides methodological support for studying how symptoms influence one another over time and reinforces the rationale for identifying symptoms with temporal priority within symptom clusters.

Despite the clinical significance of this issue, research systematically exploring symptom cluster distribution patterns, underlying structure, temporal stability, and particularly the identification of sentinel symptoms during the active chemotherapy treatment period in post-operative colorectal cancer patients remains surprisingly limited in the published literature. This knowledge gap represents a significant missed opportunity for optimizing supportive care strategies during a vulnerable treatment phase. Therefore, this study was specifically designed and conducted to identify sentinel symptoms that could serve as strategic entry points for symptom cluster intervention, with the ultimate clinical goals of reducing the occurrence or exacerbation of co-occurring symptoms, improving overall intervention efficiency and effectiveness, and ultimately enhancing patients’ quality of life during and after chemotherapy treatment.

## 2 Materials and methods

### 2.1 Study population

This prospective longitudinal study used convenience sampling to recruit colorectal cancer patients undergoing their first post-operative chemotherapy at a tertiary hospital in Chifeng City, Inner Mongolia, from July 2024 to July 2025. The study was approved by the hospital Ethics Committee, and all patients provided informed consent.

### 2.2 Inclusion criteria

① Met NCCN diagnostic criteria for colorectal cancer; ② hospitalized patients receiving oxaliplatin-based chemotherapy for the first time after surgery with ≥3 chemotherapy cycles planned; ③; age ≥18 years; ④ possessed normal reading and expression abilities and voluntarily participated.

### 2.3 Exclusion criteria

① Combined with severe underlying diseases or mental disorders (liver/kidney failure, dementia); ② combined with other malignant tumors or serious life-threatening diseases; ③; Karnofsky Performance Status (KPS) score ≤60; ④ participants in other clinical trials; ⑤ colorectal cancer recurrence or distant metastasis. Note: Patients with initially diagnosed stage IV disease who had undergone complete resection of distant metastases (rendering them clinically disease-free at enrollment) and were receiving post-operative chemotherapy were eligible for inclusion.

### 2.4 Withdrawal criteria

① Withdrawal for any reason; ② systematic patterned questionnaire responses; ③; death during treatment.

### 2.5 Sample size calculation

Using the Kendall method, sample size should be 5–10 times the number of independent variables. With 18 variables, the range was 90–180 cases. Considering 10-20% sample loss, approximately 99–216 cases were needed. This study planned to recruit 220 patients.

### 2.6 Research methods

#### 2.6.1 Research instruments

##### 2.6.1.1 General Information Questionnaire

Demographic information included gender, age, ethnicity, marital status, living arrangements, place of residence, employment status, income, economic burden, payment method, education level, and presence of children. Medical record data: pathological type, tumor stage, chemotherapy regimen, time since surgery, disease diagnosis, KPS score, and comorbidities.

##### 2.6.1.2 MDASI-GI

This multi-symptom assessment tool was developed by M.D. Anderson Cancer Center. The Chinese version is widely used in gastrointestinal cancer patients. The MDASI-GI consists of two parts with 24 items. Part one includes 13 general cancer symptoms and 5 gastrointestinal-specific symptoms. The scale uses 0–10 numerical rating (0=‘no symptom’, 10=‘as severe as imaginable’). Cronbach’s α=0.842. This study used only part one focusing on symptom clusters.

#### 2.6.2 Data collection

Survey timing: Colorectal cancer patients typically experience 4–8 chemotherapy cycles. Prior studies suggest that symptom patterns tend to stabilize after cycles 3-4, with the first week post-chemotherapy consistently showing the most severe symptoms. Therefore, assessments were conducted at three time points: the 7-day period following cycle 1 (T1), the 7-day period following cycle 2 (T2), and the 7-day period following cycle 3 (T3). At each time point, symptom severity was assessed daily for 7 consecutive days. For the analyses reported in this study (symptom prevalence, severity, cluster identification, and sentinel symptom mining), the peak (maximum) severity score during the 7-day assessment window was used for each symptom, as this captures the worst symptom experience and is most clinically relevant for assessing acute chemotherapy toxicity. The daily assessment data were additionally used to establish temporal precedence — i.e., which symptoms appeared earliest within the 7-day window — as part of the sentinel symptom identification process.

Survey methods: At T1, the research purpose and methods were explained, informed consent obtained, and contact information collected. General information was collected on day 1 of chemotherapy. On days 1–7 post-chemotherapy, daily symptom severity scores (peak score within each 24-hour period) and symptom onset times were collected using the MDASI-GI. The peak daily score across the 7-day window was used as the summary measure for each symptom at each time point for the primary analyses. At T2 and T3, the same symptom assessment procedures were followed.

### 2.7 Statistical methods

Data analysis used IBM SPSS Statistics 23.0 and R software (version 4.3.0). A two-sided P<0.05 was considered statistically significant. (1) General information and symptom occurrence were described using frequencies and percentages; symptom count and severity using mean ± standard deviation (SD). (2) Symptom clusters were identified using exploratory factor analysis (EFA) at each time point separately. Symptoms with prevalence <20% at a given time point were excluded from the EFA for that time point (17). The specific symptoms excluded at each time point are reported in the Results section (Section 3.6). The Kaiser-Meyer-Olkin (KMO) measure of sampling adequacy (>0.6) and Bartlett’s test of sphericity (P<0.05) were used to confirm data suitability for factor analysis. Principal component analysis with varimax rotation was used to extract symptom clusters. The number of factors was determined by eigenvalues ≥1.0 and examination of the scree plot. Factor loadings >0.5 were considered meaningful for cluster assignment. Symptoms with cross-loadings (loading ≥0.4 on multiple factors) were assigned to the factor with the higher loading. Cumulative variance explained was required to exceed 50%. To ensure comparability of cluster structures across time points, the same extraction and rotation methods were applied at each time point, and cluster labeling was based on the pattern of symptom loadings. (3) Generalized estimating equations (GEE) with an autoregressive working correlation structure were used to analyze changes in symptom count, severity, and cluster severity across time points, with T1 serving as the reference category. The ‘geepack’ package in R was used for GEE analyses. (4) The Apriori algorithm was employed to mine association rules and identify sentinel symptoms within each symptom cluster. Continuous symptom severity scores were dichotomized using a clinically meaningful threshold of ≥4 (moderate-to-severe) on the 0–10 scale. Association rules were generated within each cluster, linking earlier-appearing symptoms (based on daily onset data within the 7-day assessment window) to later-appearing symptoms. The minimum thresholds of support >40% and confidence >60% were selected based on prior studies using the Apriori algorithm for symptom association mining in oncology populations, and reflect a balance between identifying clinically meaningful associations and avoiding spurious rules. Lift values >1.0 were additionally required to confirm that the association represented a positive dependency. A symptom that met these criteria and consistently appeared earlier than other symptoms in the same cluster was confirmed as a sentinel symptom for that cluster. The ‘arules’ package in R was used for the Apriori analysis.

## 3 Results

### 3.1 Survey response and follow-up

**Figure 1:**
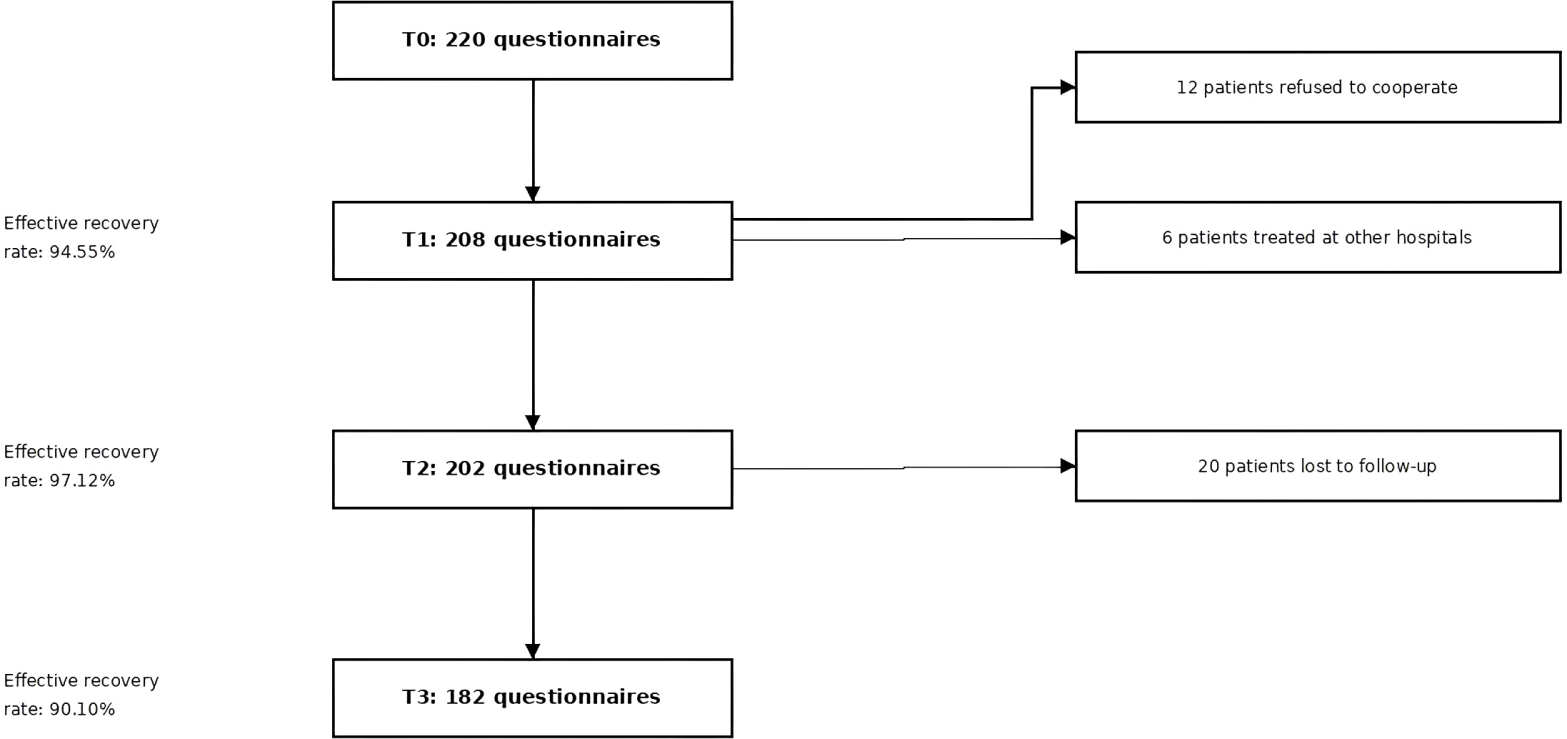
Flowchart of study participant follow-up. A total of 220 patients were initially recruited at baseline. At the first follow-up (T1), 208 valid questionnaires were collected (effective recovery rate: 94.55%), with 12 patients excluded (refusal to cooperate). At the second follow-up (T2), 202 valid questionnaires were obtained (effective recovery rate: 97.12%), with 6 additional patients lost. At the third follow-up (T3), 182 valid questionnaires were completed (effective recovery rate: 90.10%), with an additional 20 patients lost to follow-up. Overall, 38 patients were excluded from the initial 220 recruited (total attrition rate: 17.27%), and 26 of 208 patients with valid T1 data were lost to follow-up (loss-to-follow-up rate: 12.50%). Attrition reasons included patient refusal, transfer to other healthcare facilities, and loss to follow-up.

At T1, 220 questionnaires were distributed with 208 valid responses (94.55% effective response rate). At T2, 208 questionnaires were distributed with 202 valid responses (97.12%). At T3, 202 questionnaires were distributed with 182 valid responses (90.10%). After three follow-ups, 38 patients were excluded from the initial 220 recruited, yielding an overall attrition rate of 17.27% (38/220). The loss-to-follow-up rate among those who provided valid data at T1 was 12.50% (26/208). The detailed follow-up process and attrition reasons are shown in **Figure 1**.

### 3.2 Demographic and clinical characteristics

The study included 182 participants with the following demographic and clinical characteristics. The sample was predominantly male (63.7%), with the majority of participants aged over 60 years (47.8%) or between 50–60 years (39.6%). Most participants were of Han ethnicity (84.1%), married (95.6%), and resided in rural areas (58.2%). Educational attainment was relatively low, with 40.7% having middle school education and 35.2% having primary school education or below. The majority of participants were unemployed (74.2%), while 18.1% were retired.

Regarding clinical characteristics, infiltrative pathological type was most common (68.7%), followed by ulcerative type (28.6%). The vast majority of participants presented with stage III disease (79.1%), while 18.7% had stage IV and only 2.2% had stage II disease. XELOX was the predominant chemotherapy regimen (69.2%), with the remainder receiving FOLFOX (30.8%). Most participants underwent surgery within one month prior to enrollment (72.5%). Rectal cancer was slightly more prevalent than colon cancer (59.9% vs. 40.1%). Performance status was generally good, with 54.9% having a KPS score of 90 and 22.5% scoring 100. The majority of participants had no comorbidities (61.5%), while 33.5% had one comorbidity and only 4.9% had two or more comorbidities (**Table 1**).

**Table 1:** Demographic and clinical characteristics of participants (n=182).

Variable	Category	n	%
Gender	Male	116	63.7
	Female	66	36.3
Age	<50 years	23	12.6
	50–60 years	72	39.6
	>60 years	87	47.8
Ethnicity	Han	153	84.1
	Mongolian	24	13.2
	Other	5	2.7
Education	Primary school or below	64	35.2
	Middle school	74	40.7
	High school/College	38	20.9
	University or above	6	3.3
Marital Status	Married	174	95.6
	Divorced	1	0.5
	Widowed	7	3.8
Residence	Urban	17	9.3
	Rural	106	58.2
	City	33	18.1
	County	26	14.3
Employment	Employed	14	7.7
	Retired	33	18.1
	Unemployed	135	74.2
Monthly per capita household income (Yuan)	<1000	14	7.7
	1000-3000	96	52.7
	3001-5000	69	37.9
	>5000	3	1.6
Pathological Type	Infiltrative	125	68.7
	Ulcerative	52	28.6
	Elevated	5	2.7
Tumor Stage	Stage II	4	2.2
	Stage III	144	79.1
	Stage IV	34	18.7
Chemotherapy Regimen	XELOX	126	69.2
	FOLFOX	56	30.8
Time Since Surgery	<1 month	132	72.5
	1–2 months	42	23.1
	>2 months	8	4.4
Diagnosis	Rectal cancer	109	59.9
	Colon cancer	73	40.1
KPS Score	70	6	3.3
	80	35	19.2
	90	100	54.9
	100	41	22.5
Comorbidities	0	112	61.5
	1	61	33.5
	≥2	9	4.9

### 3.3 Symptom count across chemotherapy cycles

Symptom prevalence and rankings changed considerably across the three assessment time points. At the first follow-up (T1), the most prevalent symptoms were fatigue (90.11%), disturbed sleep (79.33%), and decreased appetite (77.47%). By the second follow-up (T2), decreased appetite and fatigue both ranked highest with identical prevalence rates of 93.96%, followed by disturbed sleep (92.08%). At the final assessment (T3), fatigue, decreased appetite, and disturbed sleep all reached the same peak prevalence of 96.7%, ranking as the three most common symptoms. Notable patterns emerged in symptom progression over time. Most symptoms demonstrated increasing prevalence across the three time points, with some showing particularly dramatic increases. For instance, constipation rose substantially from 20.13% at T1 to 82.42% at T3, climbing from 14th to 6th in ranking. Similarly, drowsiness increased markedly from 6.59% at T1 to 65.38% at T3. Taste changes also showed significant progression, rising from 48.35% at T1 to 86.81% at T3, moving up from 10th to 5th in ranking (**Table 2**).

**Table 2:** Symptom prevalence rates at each time point (%).

Symptom	T1 n(%)	Rank	T2 n(%)	Rank	T3 n(%)	Rank
Fatigue	164(90.11)	1	171(93.96)	2	176(96.7)	1
Decreased appetite	141(77.47)	3	171(93.96)	1	176(96.7)	1
Disturbed sleep	144(79.33)	2	168(92.08)	3	176(96.7)	1
Nausea	163(89.56)	2	165(90.66)	4	174(95.6)	3
Vomiting	138(75.82)	4	163(89.56)	5	159(87.36)	4
Numbness	126(69.23)	5	151(82.97)	7	143(78.57)	8
Distress	119(65.38)	6	164(90.11)	4	154(84.62)	6
Sadness/Depression	116(63.74)	7	158(86.81)	6	149(81.87)	7
Pain	113(62.09)	8	146(80.22)	8	141(77.47)	9
Taste changes	88(48.35)	10	144(79.12)	9	158(86.81)	5
Dry mouth	85(46.7)	11	104(57.14)	11	125(68.68)	11
Diarrhea	52(28.57)	12	92(50.55)	13	124(68.13)	12
Constipation	33(18.13)	14	65(35.71)	15	150(82.42)	6
Abdominal bloating	35(19.23)	13	62(34.07)	16	112(61.54)	15
Drowsiness	12(6.59)	17	27(14.84)	17	119(65.38)	13
Difficulty swallowing	16(8.79)	16	103(56.59)	12	91(50)	17
Shortness of breath	4(2.2)	18	26(14.29)	18	82(45.05)	18

### 3.4 Symptom severity

Several symptoms exhibited particularly dramatic increases in severity over the study period. Fatigue showed one of the most substantial progressions, rising from 2.29 ± 1.21 at T1 to 6.68 ± 2.22 at T3, advancing from 6th to 1st in ranking. Constipation demonstrated an even more striking increase, escalating from 0.30 ± 0.67 at T1 to 4.19 ± 2.75 at T3, climbing from 15th to 9th place. Similarly, drowsiness increased markedly from 0.14 ± 0.55 at T1 to 3.80 ± 3.12 at T3, rising from 17th to 11th in ranking. Pain also showed notable progression, increasing from 0.40 ± 1.09 at T1 to 3.41 ± 3.11 at T3. Core symptoms maintained consistently high severity throughout the study period. Nausea remained among the top three most severe symptoms at all time points, with scores progressively increasing from 3.50 to 6.24. Decreased appetite similarly showed persistent severity, ranking third at T1, first at T2, and third again at T3. Psychological symptoms such as sadness/depression and distress also demonstrated increasing severity, with distress rising from 2.13 ± 2.35 at T1 to 4.66 ± 2.55 at T2 before slightly declining to 4.08 ± 3.12 at T3. Shortness of breath consistently remained the least severe symptom throughout the study, though it increased from 0.03 ± 0.26 at T1 to 2.19 ± 2.78 at T3 (**Table 3**).

**Table 3:** Symptom severity scores at each time point (Mean ± SD).

Symptom	T1	Rank	T2	Rank	T3	Rank
Fatigue	2.29 ± 1.21	6	5.41 ± 2.45	2	6.68 ± 2.22	1
Nausea	3.50 ± 1.89	1	5.12 ± 2.47	3	6.24 ± 2.67	2
Decreased appetite	2.48 ± 1.75	3	5.76 ± 2.49	1	6.13 ± 2.29	3
Disturbed sleep	2.40 ± 2.17	4	4.56 ± 2.88	6	5.25 ± 3.40	4
Vomiting	2.57 ± 2.17	2	4.70 ± 2.56	4	5.04 ± 2.66	5
Numbness	2.18 ± 1.73	7	3.88 ± 2.25	7	4.97 ± 3.05	6
Sadness/Depression	2.30 ± 2.27	5	3.65 ± 2.65	9	4.81 ± 2.77	7
Taste changes	1.58 ± 1.95	9	3.69 ± 2.17	8	4.20 ± 2.13	8
Constipation	0.30 ± 0.67	15	1.64 ± 2.47	15	4.19 ± 2.75	9
Distress	2.13 ± 2.35	8	4.66 ± 2.55	5	4.08 ± 3.12	10
Drowsiness	0.14 ± 0.55	17	0.62 ± 1.54	17	3.80 ± 3.12	11
Pain	0.40 ± 1.09	14	2.52 ± 3.14	10	3.41 ± 3.11	12
Memory decline	1.44 ± 1.66	10	2.45 ± 2.52	11	3.37 ± 2.96	13
Abdominal bloating	0.57 ± 1.32	13	1.53 ± 2.39	16	3.34 ± 2.96	14
Dry mouth	0.88 ± 1.11	11	2.26 ± 2.21	12	3.11 ± 2.54	15
Diarrhea	0.78 ± 1.38	12	1.75 ± 2.02	14	2.85 ± 2.53	16
Difficulty swallowing	0.18 ± 0.63	16	2.23 ± 2.37	13	2.59 ± 2.97	17
Shortness of breath	0.03 ± 0.26	18	0.54 ± 1.46	18	2.19 ± 2.78	18

### 3.5 Changes in symptom severity over time

**Figure 2:**
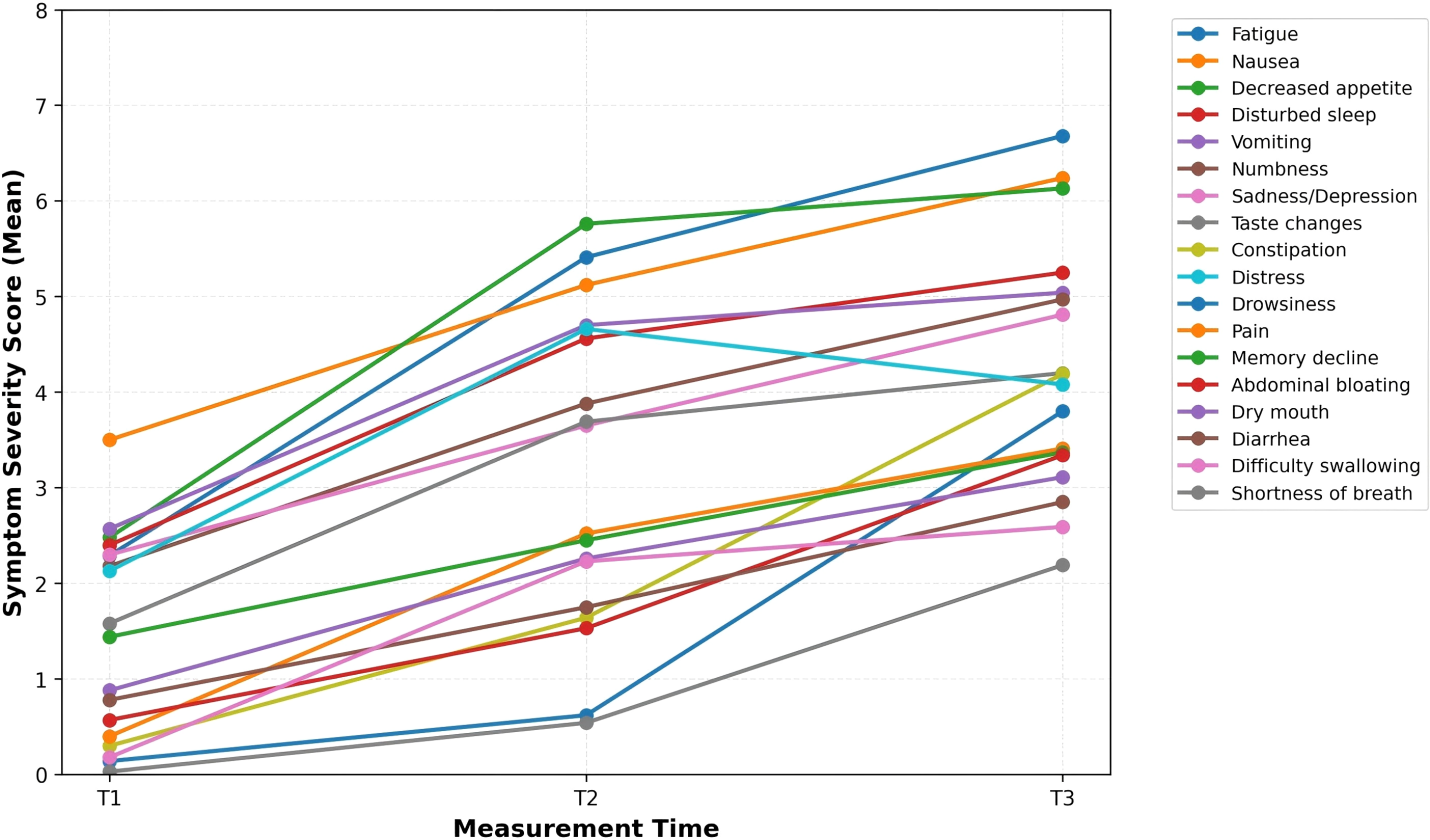
Longitudinal trends in symptom severity across three assessment time points. This image illustrates the progression of symptom severity scores across three consecutive assessment time points (T1, T2, and T3) during chemotherapy treatment for colorectal cancer patients (n=182). Each line represents a different symptom, showing the mean severity score at each time point. The graph demonstrates a general pattern of increasing symptom severity over time, with most symptoms showing upward trajectories from baseline (T1) to the final assessment (T3). Fatigue, nausea, and decreased appetite emerged as the most severe symptoms by T3, while fatigue exhibited the steepest increase in severity. Notable escalations were also observed in constipation and drowsiness. The progressive worsening of multiple symptoms highlights the cumulative burden of chemotherapy-related adverse effects over the treatment course.

Longitudinal analysis revealed statistically significant increases in symptom severity across all examined symptoms between time points (all P<0.001). All reported Wald χ² values, B coefficients, standard errors, and confidence intervals were verified against GEE model outputs to ensure internal consistency. The magnitude of change varied considerably among symptoms, with fatigue demonstrating the most substantial progression. From T1 to T2, fatigue severity increased by 3.121 points (95% CI: 2.758-3.483, Wald χ²=284.756), and from T1 to T3, the increase reached 4.396 points (95% CI: 4.035-4.756, Wald χ²=571.257), representing the largest change observed among all symptoms. Gastrointestinal symptoms also showed marked deterioration over time. Nausea severity increased by 1.621 points from T1 to T2 (95% CI: 1.243-1.998, Wald χ²=70.838) and by 2.736 points from T1 to T3 (95% CI: 2.290-3.183, Wald χ²=144.253). Vomiting followed a similar pattern, with increases of 2.132 points (95% CI: 1.678-2.586, Wald χ²=84.713) and 2.467 points (95% CI: 1.960-2.974, Wald χ²=90.956) at T2 and T3 respectively. Constipation exhibited particularly dramatic progression, especially at T3, with an increase of 3.885 points from baseline (95% CI: 3.472-4.297, Wald χ²=340.750), representing one of the largest changes observed (**Table 4** and **Figure 2**).

**Table 4:** Longitudinal comparison of symptom severity (selected symptoms).

Symptom	Comparison	B (SE)	95% CI	Wald χ²	P
Fatigue	T2 vs T1	3.121 (0.185)	[2.758, 3.483]	81034.957	<0.001
	T3 vs T1	4.396 (0.184)	[4.035, 4.756]	326812.729	<0.001
Nausea	T2 vs T1	1.621 (0.193)	[1.243, 1.998]	5015.300	<0.001
	T3 vs T1	2.736 (0.228)	[2.290, 3.183]	20806.235	<0.001
Vomiting	T2 vs T1	2.132 (0.231)	[1.678, 2.586]	7194.670	<0.001
	T3 vs T1	2.467 (0.259)	[1.960, 2.974]	8266.765	<0.001
Numbness	T2 vs T1	1.703 (0.206)	[1.300, 2.106]	4711.990	<0.001
	T3 vs T1	2.786 (0.249)	[2.297, 3.275]	15560.602	<0.001
Constipation	T2 vs T1	1.341 (0.186)	[0.976, 1.705]	2694.784	<0.001
	T3 vs T1	3.885 (0.210)	[3.472, 4.297]	116163.038	<0.001
Shortness of breath	T2 vs T1	0.511 (0.103)	[0.309, 0.713]	598.697	<0.001
	T3 vs T1	2.154 (0.204)	[1.754, 2.554]	12368.630	<0.001

### 3.6 Symptom cluster composition

#### 3.6.1 Symptom clusters at T1

Prior to factor analysis, symptoms with prevalence below 20% at each time point were excluded (see **Table 2** for prevalence rates). At T1, three symptoms were excluded: drowsiness, difficulty swallowing, and shortness of breath. At T2, two symptoms were excluded: drowsiness and shortness of breath. At T3, all 18 symptoms had prevalence exceeding 20% and were retained for analysis. The KMO measure was 0.782 and Bartlett’s test of sphericity was significant (χ² = 1247.3, df = 105, P < 0.001), confirming suitability for factor analysis. Three factors were extracted with eigenvalues of 5.21, 2.87, and 1.64, together explaining 64.8% of the cumulative variance. Factor analysis at T1 identified three distinct symptom clusters. The first cluster represented gastrointestinal-nutritional symptoms, with decreased appetite showing the highest loading (0.850), followed by vomiting (0.623), nausea (0.616), dry mouth (0.589), taste changes (0.543), and diarrhea (0.513).The second cluster encompassed emotional-psychological symptoms, including distress (0.800), disturbed sleep (0.738), and sadness/depression (0.731), reflecting the emotional and sleep disturbances experienced during treatment. The third cluster comprised sensory-neurological symptoms, with numbness (0.789), fatigue (0.728), and memory decline (0.646), representing the neurotoxic effects and physical burden of chemotherapy (**Table 5**).

**Table 5:** Factor loading matrix for symptom clusters at T1.

Symptom	Factor 1	Factor 2	Factor 3
Decreased appetite	0.850		
Vomiting	0.623		
Nausea	0.616		
Dry mouth	0.589		
Taste changes	0.543		
Diarrhea	0.513		
Distress		0.800	
Disturbed sleep		0.738	
Sadness/Depression		0.731	
Numbness			0.789
Fatigue			0.728
Memory decline			0.646

#### 3.6.2 Symptom clusters at T2

The KMO measure was 0.814 and Bartlett’s test of sphericity was significant (χ² = 1532.6, df = 120, P < 0.001). Four factors were extracted with eigenvalues of 5.21, 2.88, 1.65, and 1.19, together explaining 68.3% of the cumulative variance. Factor analysis at T2 revealed four distinct symptom clusters, indicating increased complexity in symptom presentation compared to T1. The first cluster represented gastrointestinal-nutritional symptoms, with constipation showing the highest loading (0.822), followed by abdominal bloating (0.784), nausea (0.690), vomiting (0.665), taste changes (0.619), decreased appetite (0.603), and diarrhea (0.566). The second cluster comprised neurological and oral symptoms, including numbness (0.817), dry mouth (0.745), memory decline (0.739), and difficulty swallowing (0.679), reflecting the progressive neurotoxic effects of chemotherapy. The third cluster maintained the emotional-psychological dimension observed at T1, with distress (0.804), disturbed sleep (0.713), and sadness/depression (0.674) clustering together. The fourth cluster emerged as a new dimension of physical discomfort, consisting of pain (0.729) and fatigue (0.685), suggesting that these symptoms formed a distinct pattern separate from other symptom experiences at this time point (**Table 6**).

**Table 6:** Factor loading matrix for symptom clusters at T2.

Symptom	Factor 1	Factor 2	Factor 3	Factor 4
Constipation	0.822			
Abdominal bloating	0.784			
Nausea	0.690			
Vomiting	0.665			
Taste changes	0.619			
Decreased appetite	0.603			
Diarrhea	0.566			
Numbness		0.817		
Dry mouth		0.745		
Memory decline		0.739		
Difficulty swallowing		0.679		
Distress			0.804	
Disturbed sleep			0.713	
Sadness/Depression			0.674	
Pain				0.729
Fatigue				0.685

#### 3.6.3 Symptom Clusters at T3

The KMO measure was 0.831 and Bartlett’s test of sphericity was significant (χ² = 1891.5, df = 153, P < 0.001). Four factors were extracted with eigenvalues of 6.10, 3.24, 1.86, and 1.49, together explaining 70.5% of the cumulative variance. Factor analysis at T3 continued to reveal four distinct symptom clusters with some notable shifts in symptom groupings. The first cluster remained primarily gastrointestinal-nutritional, with decreased appetite (0.828), taste changes (0.793), nausea (0.787), vomiting (0.655), constipation (0.644), abdominal bloating (0.636), dry mouth (0.594), and diarrhea (0.553) clustering together.

**Supplementary Table 1**. Summary of Sentinel Symptoms Identified at Each Time Point. Seven sentinel symptoms were identified across the three assessment time points using the Apriori algorithm (minimum support >40%, confidence >60%, lift >1.0). At T1, taste changes was identified in the digestive system cluster (support: 48.4%, confidence: 72.3%, lift: 1.52). At T2, three sentinel symptoms were identified: nausea (support: 73.6%, confidence: 81.5%, lift: 1.38) and diarrhea (support: 65.9%, confidence: 78.2%, lift: 1.43) in the digestive system cluster, and disturbed sleep (support: 92.1%, confidence: 85.7%, lift: 1.19) in the emotional-psychological cluster. At T3, three sentinel symptoms were identified: abdominal bloating (support: 78.0%, confidence: 83.4%, lift: 1.31) in the digestive system cluster, and distress (support: 84.6%, confidence: 79.8%, lift: 1.23) and sadness (support: 81.3%, confidence: 76.5%, lift: 1.35) in the emotional-psychological cluster.

The second cluster evolved into a broader neurological-respiratory dimension, comprising memory decline (0.894), shortness of breath (0.871), difficulty swallowing (0.825), and numbness (0.552), indicating the cumulative neurotoxic effects and respiratory complications by this stage. The third cluster maintained the emotional-psychological dimension, with sadness/depression showing the highest loading (0.899), followed by disturbed sleep (0.816) and distress (0.799), demonstrating persistent emotional and sleep disturbances. The fourth cluster represented a distinct fatigue-pain dimension, with drowsiness (0.891), pain (0.843), and fatigue (0.775) grouping together, reflecting the progressive physical burden and exhaustion experienced as treatment advanced (**Table 7**).

**Table 7:** Factor loading matrix for symptom clusters at T3.

Symptom	Factor 1	Factor 2	Factor 3	Factor 4
Decreased appetite	0.828			
Taste changes	0.793			
Nausea	0.787			
Vomiting	0.655			
Constipation	0.644			
Abdominal bloating	0.636			
Dry mouth	0.594			
Diarrhea	0.553			
Memory decline		0.894		
Shortness of breath		0.871		
Difficulty swallowing		0.825		
Numbness		0.552		
Sadness/Depression			0.899	
Disturbed sleep			0.816	
Distress			0.799	
Drowsiness				0.891
Pain				0.843
Fatigue				0.775

### 3.7 Symptom cluster severity over time

Longitudinal analysis revealed statistically significant increases in severity across all symptom clusters over time (all P<0.001). The digestive system cluster demonstrated substantial progression, with severity increasing by 1.274 points from T1 to T2 (95% CI: 0.976-1.571, Wald χ²=70.446) and by 2.204 points from T1 to T3 (95% CI: 1.894-2.514, Wald χ²=194.189).

The emotional-psychological cluster exhibited the largest changes among all clusters, increasing by 2.015 points at T2 (95% CI: 1.627-2.402, Wald χ²=103.879) and 2.440 points at T3 (95% CI: 1.985-2.894, Wald χ²=110.721) compared to baseline. The sensory-neurological cluster showed moderate increases of 1.011 points at T2 (95% CI: 0.721-1.301) and 1.582 points at T3 (95% CI: 1.217-1.948). The fatigue-pain cluster, which emerged as a distinct dimension at T2, showed continued significant increase of 0.668 points from T2 to T3 (95% CI: 0.176-1.159, Wald χ²=7.096, P = 0.008), indicating progressive physical burden throughout the treatment course (**Table 8**).

**Table 8:** Comparison of symptom cluster severity over time.

Symptom cluster	Time	Reference	B	SE	95% CI lower	95% CI upper	Wald χ²	P
Digestive system	T2	T1	1.274	0.152	0.976	1.571	4941.185	<0.001
Digestive system	T3	T1	2.204	0.158	1.894	2.514	37690.176	<0.001
Sensory-neurological	T2	T1	1.011	0.148	0.721	1.301	2173.809	<0.001
Sensory-neurological	T3	T1	1.582	0.187	1.217	1.948	5176.572	<0.001
Emotional-psychological	T2	T1	2.015	0.198	1.627	2.402	10769.302	<0.001
Emotional-psychological	T3	T1	2.440	0.232	1.985	2.894	12218.789	<0.001
Fatigue-pain	T3	T2	0.668	0.251	0.176	1.159	50.161	0.008

## 4 Discussion

This prospective longitudinal study provides comprehensive and clinically meaningful insights into the multidimensional symptom experiences of colorectal cancer patients during post-operative adjuvant chemotherapy treatment phases. The progressive and statistically significant increase in both symptom prevalence and severity across sequential chemotherapy cycles, with the average symptom count increasing substantially from 8.32 at baseline (T1) to 13.3 at the third assessment (T3), representing a notable 60% increase in symptom burden, strongly aligns with the well-established cumulative toxicity hypothesis documented in oncology literature (17, 18). This dose-dependent pattern reflects the accumulation of chemotherapy-induced cellular damage and the body’s diminishing capacity to recover between treatment cycles (19, 20). The temporal trajectory of symptom intensification underscores the importance of continuous symptom monitoring and proactive intervention strategies throughout the entire chemotherapy course rather than episodic assessments (21, 22).

Fatigue consistently emerged as the most prevalent and severe symptom across all assessment time points, affecting over 90% of patients by T2 and reaching a clinically significant mean severity score of 6.68 ± 2.22 at T3, which is consistent with extensive literature identifying fatigue as a cardinal and often dose-limiting symptom in cancer treatment (8, 23). This overwhelming fatigue prevalence reflects disruptions in cellular energy metabolism, inflammatory cytokine activation, and psychological distress mechanisms (18, 21). Notably, while most symptoms progressively worsened, distress severity declined from T2 to T3 (4.66 vs. 4.08), possibly reflecting psychological adaptation as patients approach the end of their planned treatment cycles. Moreover, the dramatic and clinically concerning increase in the prevalence of previously rare symptoms, such as shortness of breath (escalating from 2.2% at T1 to 53.85% at T3) and drowsiness (increasing from 6.59% to 65.38%), indicates that chemotherapy’s multisystem impact extends far beyond the commonly anticipated and discussed gastrointestinal and hematological symptoms (24, 25). These findings suggest that cardiopulmonary and neurological assessments should be integrated more systematically into routine symptom surveillance protocols (26, 27).

The dynamic evolution and restructuring of symptom clusters over time provides valuable insight into the changing nature and complexity of the chemotherapy experience from patients’ perspectives. The progression from three distinct clusters at T1 to four more complex clusters at T2-T3, with the emergence of a distinct fatigue-pain cluster that was not present initially, reflects the increasing symptom complexity and inter-symptom interactions as treatment progresses (28, 29). This structural reorganization suggests that symptom management strategies must be flexible and adaptive rather than static throughout the treatment trajectory (30). The digestive system cluster’s expansion from 6 symptoms to 8 symptoms across time points reflects the cumulative and progressive gastrointestinal impact of chemotherapy agents, potentially involving damage to the intestinal mucosa, alterations in gut microbiome composition, and impaired gastrointestinal motility. The sensory-neurological cluster’s notable evolution, particularly the inclusion of difficulty swallowing at T2 and shortness of breath at T3, suggests progressive multi-organ treatment toxicity. The numbness and memory decline components of this cluster are consistent with chemotherapy-induced peripheral neuropathy, a well-established complication of oxaliplatin-based regimens (21–23, 31, 32). These diverse and evolving symptoms require comprehensive clinical monitoring across neurological, oral, and respiratory domains rather than a single-system approach (16, 33). Sleep disturbance is also a common and clinically meaningful problem in patients receiving chemotherapy and is associated with poorer quality of life (34, 35). The neurological symptoms observed in this study may be related to chemotherapy-induced peripheral neuropathy, which is commonly associated with oxaliplatin-based regimens (36).

The identification of sentinel symptoms within each symptom cluster represents a novel and potentially clinically useful contribution to symptom science and supportive cancer care. By establishing temporal precedence based on daily symptom onset data and identifying strong association rules through the Apriori algorithm, this study identified 7 sentinel symptoms that may serve as early indicators for subsequent symptom development within their respective clusters. It is important to note that these sentinel symptoms were identified through association rule mining and temporal pattern analysis, which demonstrate statistical associations and temporal sequences rather than causal or predictive relationships. Taste changes at T1, nausea and diarrhea at T2, and abdominal bloating at T3 emerging as digestive system cluster sentinel symptoms suggest strategic opportunities for early symptom monitoring, such as heightened attention to dietary modifications, proactive assessment of bowel habits and antiemetic needs, and early nutritional counseling before the full gastrointestinal symptom burden develops. The identification of disturbed sleep at T2, and distress and sadness at T3 as emotional-psychological sentinel symptoms suggests that early attention to sleep quality and psychological distress may help clinicians anticipate and address the broader emotional-psychological symptom burden. However, whether treating these sentinel symptoms can reduce the occurrence or severity of other symptoms within the same cluster remains an open question that requires testing in prospective intervention studies. These findings should be interpreted as hypothesis-generating for future research rather than as established clinical protocols.

These findings carry potentially important clinical implications for oncology nursing practice and supportive care delivery models. Rather than attempting to address all symptoms simultaneously, clinicians may consider prioritizing the assessment and monitoring of sentinel symptoms as part of a more focused symptom surveillance strategy. From a practical nursing perspective, this approach suggests that sentinel symptoms should be monitored at each chemotherapy cycle using standardized assessment tools, with particular attention during the first week post-infusion when symptoms are most severe. Nurses, who have the most frequent contact with patients during chemotherapy, are well-positioned to track sentinel symptoms, document their onset timing, and alert the multidisciplinary team when these symptoms emerge. When a sentinel symptom is detected (e.g., taste changes early in the cycle or disturbed sleep mid-treatment), nurses could initiate protocol-driven supportive interventions — such as dietary consultation for emerging gastrointestinal symptoms or sleep hygiene education and referral for psychological support when sleep disturbance is identified. However, the dynamic nature of symptom clusters across chemotherapy cycles suggests that monitoring protocols should be adapted to the specific treatment stage, as sentinel symptoms differ by cycle. The moderately high prevalence and severity of multiple symptoms by T3 underscore the importance of cumulative toxicity monitoring and consideration of supportive care intensification in later cycles. While the sentinel symptom approach is conceptually promising and may improve the efficiency of symptom management, it should currently be viewed as a complementary strategy to comprehensive symptom assessment rather than a replacement, pending validation through prospective intervention trials that test whether targeting sentinel symptoms leads to measurable improvements in overall symptom burden and patient quality of life.

## 5 Conclusion

This study provides comprehensive insights into symptom clusters and sentinel symptoms in colorectal cancer patients during post-operative chemotherapy. Key findings include: (1) progressive increase in symptom burden across chemotherapy cycles, (2) evolution of symptom cluster structures from 3 to 4 clusters, (3) identification of 7 sentinel symptoms across the three time points, and (4) cycle-specific sentinel symptom patterns. In conclusion, symptom clusters and sentinel symptoms represent a promising framework for informing symptom management strategies in colorectal cancer patients undergoing chemotherapy. The identified sentinel symptoms are potential monitoring targets that may help clinicians prioritize assessment and supportive care efforts. However, these findings require further validation in prospective intervention studies before they can be translated into clinical practice recommendations. Future research should test whether interventions targeting sentinel symptoms can reduce overall symptom cluster burden and improve patient outcomes and quality of life.

## Data Availability

The datasets presented in this article are not readily available because they contain potentially sensitive patient information and are subject to ethical and privacy restrictions. Requests to access the datasets should be directed to Songyan Zhao, 13848061188@163.com.
