# Supplementary material for: Symptom clusters and sentinel symptoms in colorectal cancer patients during post-operative chemotherapy
Source: Front Oncol. 2026 Jun 30;16:1808713. doi: 10.3389/fonc.2026.1808713 (PMC13364570; doi:10.3389/fonc.2026.1808713)
Supplement: Supplementary file 1 [file Table1.docx]

**Supplementary Table 1. Summary of Sentinel Symptoms Identified at Each Time Point**

Seven sentinel symptoms were identified across three assessment time points (T1, T2, T3) using the Apriori algorithm (minimum support >40%, confidence >60%, lift >1.0). Sentinel symptoms were confirmed based on meeting all three thresholds and demonstrating temporal precedence (appearing earliest) within their respective symptom cluster during the 7-day post-chemotherapy assessment window.

| **Time Point** | **Symptom Cluster** | **Sentinel Symptom** | **Support** | **Confidence** | **Lift** | **Temporal Criterion** |
| --- | --- | --- | --- | --- | --- | --- |
| **T1** | Gastrointestinal-Nutritional | **Taste Changes** | 48.4% | 72.3% | 1.52 | Appeared earliest (day 1–2 post-chemotherapy) within the cluster assessment window |
| **T2** | Gastrointestinal-Nutritional | **Nausea** | 73.6% | 81.5% | 1.38 | Appeared earliest among cluster symptoms; preceded taste changes and constipation |
|  | Gastrointestinal-Nutritional | **Diarrhea** | 65.9% | 78.2% | 1.43 | Appeared concurrently with nausea; both met temporal precedence criteria |
|  | Emotional-Psychological | **Disturbed Sleep** | 92.1% | 85.7% | 1.19 | Appeared earliest within the emotional-psychological cluster at T2 |
| **T3** | Gastrointestinal-Nutritional | **Abdominal Bloating** | 78.0% | 83.4% | 1.31 | Appeared earliest within the digestive cluster at T3 |
|  | Emotional-Psychological | **Distress** | 84.6% | 79.8% | 1.23 | Appeared earlier than sadness/depression within the emotional-psychological cluster |
|  | Emotional-Psychological | **Sadness/Depression** | 81.3% | 76.5% | 1.35 | Co-occurred with distress; met temporal precedence relative to other cluster symptoms |

*Abbreviations: T1, 7-day period following the 1st chemotherapy cycle; T2, 7-day period following the 2nd chemotherapy cycle; T3, 7-day period following the 3rd chemotherapy cycle. Support: proportion of patients in the cluster exhibiting the sentinel symptom at moderate-to-severe level (≥4/10). Confidence: proportion of patients with the sentinel symptom who subsequently developed other cluster symptoms. Lift: ratio of observed to expected co-occurrence (>1.0 indicates positive association). Temporal criterion: based on daily symptom onset data within the 7-day post-chemotherapy assessment window.*
